# First Food and Drug Administration Cleared Thin-Film Electrode for Intracranial Stimulation, Recording, and Monitoring of Brain Activity—Part 1: Biocompatibility Testing

**DOI:** 10.3389/fnins.2022.876877

**Published:** 2022-04-29

**Authors:** Aura Kullmann, Debra Kridner, Steve Mertens, Mark Christianson, Dave Rosa, Camilo A. Diaz-Botia

**Affiliations:** NeuroOne Medical Technologies Corporation, Eden Prairie, MN, United States

**Keywords:** epilepsy, polyimide, strips and grids, cytotoxicity, genotoxicity, sensitization and irritation

## Abstract

Subdural strip and grid invasive electroencephalography electrodes are routinely used for surgical evaluation of patients with drug-resistant epilepsy (DRE). Although these electrodes have been in the United States market for decades (first FDA clearance 1985), their fabrication, materials, and properties have hardly changed. Existing commercially available electrodes are made of silicone, are thick (>0.5 mm), and do not optimally conform to brain convolutions. New thin-film polyimide electrodes (0.08 mm) have been manufactured to address these issues. While different thin-film electrodes are available for research use, to date, only one electrode is cleared by Food and Drug Administration (FDA) for use in clinical practice. This study describes the biocompatibility tests that led to this clearance. Biocompatibility was tested using standard methods according to International Organization for Standardization (ISO) 10993. Electrodes and appropriate control materials were bent, folded, and placed in the appropriate extraction vehicles, or implanted. The extracts were used for *in vitro* and *in vivo* tests, to assess the effects of any potential extractable and leachable materials that may be toxic to the body. *In vitro* studies included cytotoxicity tested in L929 cell line, genotoxicity tested using mouse lymphoma assay (MLA) and Ames assay, and hemolysis tested in rabbit whole blood samples. The results indicated that the electrodes were non-cytotoxic, non-mutagenic, non-clastogenic, and non-hemolytic. *In vivo* studies included sensitization tested in guinea pigs, irritation tested in rabbits, acute systemic toxicity testing in mice, pyrogenicity tested in rabbits, and a prolonged 28-day subdural implant in sheep. The results indicated that the electrodes induced no sensitization and irritation, no weight loss, and no temperature increase. Histological examination of the sheep brain tissue showed no or minimal immune cell accumulation, necrosis, neovascularization, fibrosis, and astrocyte infiltration, with no differences from the control material. In summary, biocompatibility studies indicated that these new thin-film electrodes are appropriate for human use. As a result, the electrodes were cleared by the FDA for use in clinical practice [510(k) K192764], making it the first thin-film subdural electrode to progress from research to clinic. Its readiness as a commercial product ensures availability to all patients undergoing surgical evaluation for DRE.

## Introduction

Drug-resistant epilepsy (DRE), defined as the occurrence of uncontrolled seizures despite two tolerated and appropriately chosen antiepileptic drugs used either in combination or as monotherapies, affects 30% of more than 50 million patients with epilepsy worldwide (Chen et al., 2018). For these patients, the available surgical treatments include surgical resection of the tissue suspected of generating seizures (epileptogenic zone, EZ), ablation, or neuromodulation. The success of these surgical treatments critically depends on the identification and precise localization of the EZ and its relationship with cortical areas involved in daily function (e.g., language, vision, and movement areas). Subdural electrodes are routinely used for this purpose. These electrodes are placed via a craniotomy under the dura and used to monitor and record brain activity and stimulate for a prolonged time (less than 30 days) to localize the EZ and determine if resection is feasible. Existing subdural electrodes are bulky, thick (approximately 0.5 mm), and do not conform well to the brain gyrations (Tong et al., 2020). The consequences of these properties negatively impact the complication rates, signal quality, and tissue’s immune response (Araki et al., 2006; Mocco et al., 2006; Van Gompel et al., 2008; Fong et al., 2012; Nagahama et al., 2018; Tong et al., 2020; Carnicer-Lombarte et al., 2021).

New thin-film electrodes have been manufactured by NeuroOne Medical Technologies Corporation (Eden Prairie, MN, United States) to address these issues. These electrodes are made of polyimide as a substrate with platinum contacts. They are 0.08 mm thick, which is about seven times thinner than the existing silicone electrodes, and very light (0.05 g including the tail) (Kullmann et al., 2021). For any new device intended for brain implant that would be commercialized in the United States, biocompatibility testing is a requirement of the USA’s FDA to demonstrate device safety. Biocompatibility is defined as the ability of a medical device or material to perform with an appropriate host response in a specific application (definition per ISO 10993:2018, Biological Evaluation of Medical Devices). The testing determines whether the device materials contain any toxic, leachable, or diffusible substances that can cause local or systemic responses or can be absorbed into the circulatory system and/or cerebrospinal fluid (CSF), evaluates the potential for systemic toxicity, mutagenesis, and tissue’s immunological response to implanted materials. The characterization consists of a comprehensive battery of *in vitro* and *in vivo* tests, performed following standards provided by the ISO 10993. The battery of tests is determined by factors including the device type, intended use, duration of patient contact, and nature of body contact (e.g., tissue/blood). For the thin-film subdural electrodes investigated here, the battery consisted of cytotoxicity, genotoxicity, hemolysis, sensitization, irritation, acute systemic toxicity, pyrogenicity, and subdural implantation.

Cytotoxicity evaluates the effect of leachable and/or diffusible substances from a test article on the morphology of mammalian cells. Genotoxicity determines whether a test material can induce either point mutations or clastogenic events, which have the potential for cancer. Hemolysis evaluates the effects of blood-contacting materials on blood and/or blood components (e.g., activation of the complement system, activation of platelets, thrombosis, embolism, or another cell injury). Sensitization test determines the sensitizing activity and the potential of a test article to cause a delayed hyper-sensitivity reaction by exposing the animals to the test article and evaluating the sensitization reactions (e.g., erythema and/or edema) (Magnusson and Kligman, 1969; Schlede and Eppler, 1995). The irritation test evaluates whether the test article can cause local irritation by applying or dosing the test article extracts directly to the animal and evaluating irritation reactions (e.g., erythema and/or edema). Acute systemic toxicity evaluates the potential of a test article to cause adverse effects distant to the entry point by dosing the animals intravenously and/or intraperitoneally with test article extracts, and monitoring for various signs of toxicity at different time intervals (Strickland et al., 2018). Pyrogenicity determines if the test article extracts can cause a febrile response in animals (Borton and Coleman, 2018). The implantation test assesses the local pathological effects on living tissue induced by the test article when surgically implanted for an extended duration of time, by using gross and microscopic examination of the exposed tissue.

Given the importance of biocompatibility testing for new devices, here, we describe the testing results for the first FDA cleared thin-film polyimide subdural electrode.

## Materials and Methods

### Facilities

Biocompatibility tests were performed under good laboratory practices (GLP) guidelines by standard operating procedures and following standard protocols, at three Contract Research Organizations (CROs), WuXI AppTec laboratories (St Paul, MN, United States), American Preclinical Services [APS, now part of North American Science Associates, Inc (NAMSA); Minneapolis, MN, United States], and NAMSA (Northwood, OH, United States). The WuXI AppTec, APS, and NAMSA are American Association for Accreditation of Laboratory Animal Care (AAALAC) accredited and GLP-compliant CROs. The APS is ISO 17025 accredited, and United States Department of Agriculture (USDA) registered facility. All animal procedures were approved by the Institutional Animal Care and Use Committee (IACUC) of APS, WuXI AppTec, or NAMSA.

### Test Article

As test articles, all tests utilized subdural strip and grid electrodes manufactured by NeuroOne Medical Technologies Corporation (Eden Prairie, MN). These thin-film electrodes (0.08 mm thickness) were made of polyimide as the substrate with 3-mm diameter platinum contacts, spaced at 10 mm center to center. Unless specified, the tests used two contacts (1 × 2) strip electrodes with a surface area of 19 cm^2^/electrode. The preparation, extraction vehicle(s), and positive and negative controls are described below for each test. Unless specified, all reagents, buffers, and chemicals were purchased from Sigma-Aldrich (St. Louis, MO, United States).

### Assays

#### Cytotoxicity (Per ISO 10993-5)

##### Test and Control Article Preparation

The test articles, consisting of electrodes (*n* = 2; total surface area 38 cm^2^) were bent, folded, and placed into an extraction vessel at a ratio of 6 cm^2^/1 ml of extraction vehicle. A negative, positive, and cell control were run in parallel with the test article. The negative control was United States Pharmacopeia High-Density Polyethylene (USP HDPE), known to be non-toxic under the test conditions, and was prepared at a ratio of 3 cm^2^/1 ml of extraction vehicle. The positive control was polyurethane film containing 0.1% zinc diethyldithiocarbamate (ZDEC), known to be toxic under the test conditions, and was prepared at a ratio of 3 cm^2^ to 1 ml of extraction vehicle. A cell control, Eagle’s minimal essential medium (E-MEM) supplemented with 5% (v/v) fetal bovine serum (FBS), was incubated in parallel with the test sample and controls. In test article, positive and negative controls were extracted in E-MEM + 5% FBS for 24 ± 2 h at 37 ± 1°C. All extracts were added in triplicates to the cell cultures.

##### Cell Cultures

The L929 cell line derived from murine fibroblasts was obtained from the American Type Culture Collection (ATCC CCL-1). Cells were grown as monolayers at 37 ± 1°C in 5 ± 1% CO_2_, in E-MEM + 5% FBS and 2-mM L-glutamine, 10 mM HEPES, 0.01 mg/ml vancomycin, 0.01 mg/ml gentamicin, 1% of 1,000 units/ml penicillin, and 1% of 2.50 g/ml amphotericin B.

##### Testing

Cell media was replaced with 1 ml of extract, positive or negative control media, and cultures were evaluated for cytotoxic effects at 24, 48, and 72 ± 4 h. The cell layer reactivity was scored on a scale of 0 to 4 for abnormal cell morphology and cellular degeneration, which included lysis, crenation, plaques, and excessive rounding of cells. According to ISO 10993-5 guidelines, the test scores are defined as follows: 0 = discrete intracytoplasmic granules; no cell lysis, no reduction of cell growth; 1 = not more than 20% of the cells are round, loosely attached, and without intracytoplasmic granules, or showed changes in morphology; occasional lysed cells are present; only slight growth inhibition observable; 2 = not more than 50% of the cells are round, devoid of intracytoplasmic granules, no extensive cell lysis; not more than 50% growth inhibition observable; 3 = not more than 70% of the cell layers contain rounded cells or are lysed; cells layers not destroyed, but more than 50% growth inhibition observable; and 4 = nearly complete or complete destruction of the cell layers. Test articles scoring “0,” “1,” or “2” are considered “non-cytotoxic.” Test articles scoring “3” or “4” are considered “cytotoxic.”

#### Genotoxicity—Mouse Lymphoma Assay (Per ISO 10993-3)

The MLA quantifies genetic alterations involving the thymidine kinase (Tk) gene (Lloyd and Kidd, 2012). The test exposes the L5178Y mouse lymphoma Tk^±^ cell line to the test article or extracts of the test article and evaluates forward mutations at the thymidine kinase (TK) locus, assayed by colony growth of L5178Y cells in the presence of trifluorothymidine (TFT). TK is an enzyme that allows cells to salvage thymidine from the surrounding medium for DNA synthesis. If thymidine analogs, such as TFT, are included in the growth medium, the analogs are phosphorylated *via* the TK pathway and cause cellular death by inhibiting DNA synthesis. Cells that are heterozygotes at the TK locus (TK^±^) may undergo a single-step forward mutation to the TK^–/–^ genotype in which little or no TK activity remains. These mutants are as viable as the heterozygotes in a normal medium because DNA synthesis proceeds by *de novo* synthesis pathways that do not involve thymidine as an intermediate. TK^–/–^ mutants cannot utilize toxic analogs of thymidine. Cells that may grow to form colonies in the presence of TFT are therefore assumed to have mutated, either spontaneously or as a result of exposure to the test article, at the TK^±^ locus. Mutation frequency (MF) is estimated by comparing the cloning efficiency of the cells in a culture medium without the selective agent. Mutagenic activity is then determined by treating cultures with different concentrations of a test article and examining the potential for concentration-related increases in MF.

##### Test Article Preparation

Electrodes (*n* = 4; total surface area 76 cm^2^) were bent, folded, and placed into an extraction vessel at a ratio of 6 cm^2^/1 ml of extraction vehicles for 72 ± 2 h at 50 ± 2°C. The vehicles were 0.9% normal saline (NS) or DMSO.

##### Cell Cultures

The L5178Y TK^±^ mouse lymphoma cell line was obtained from the European Collection of Cell Cultures (ECACC 12080201), and maintained in RPMI-1640, supplemented with 10% heat-inactivated horse serum (HIHS), 1% L-glutamine, 1% sodium pyruvate, 1% penicillin/streptomycin, and 1% Pluronic #F68 acid, at 37 ± 1°C in 5 ± 1% CO_2_. The treatment media consisted of RPMI-1640 medium with 5% HIHS. The cloning medium consisted of the preceding medium with up to 20% HIHS, without Pluroni #F68 acid, and with the addition of agar to achieve a semisolid state.

##### Metabolic Activation System

The S9 fraction (Moltox, Boone, NC) was added to the core reaction mixture at a ratio of 0.3 ml S9 to 0.7 ml core solution, resulting in a final S9 concentration of 3% v/v in culture. One milliliter of this solution was then added to 9 ml of culture media.

##### Testing

This test was conducted under three treatment conditions as follows: 4-h treatment in the presence of an exogenous mammalian activation system (+S9), 4-h treatment in the absence of exogenous mammalian activation (–S9), and a 24-h treatment in the absence of exogenous mammalian activation (for the detection of slower acting mutagens). The L5178Y TK ± cells (final cell concentration of 6 × 10^5^ cells/ml in 10 ml; 6 × 10^6^ total cells) were tested in triplicate at one dose level along with appropriate vehicle and positive controls in the presence and absence of metabolic activation. The negative control was the extracted vehicle controls ± S9 and was used to determine spontaneous mutant frequencies and cloning efficiency. The positive control, methyl methanesulfonate (MMS), was added at a final concentration of 10 μg/ml for the low dose, and 15 μg/ml for the high dose in the portion of the assay performed without activation. Cyclophosphamide (CP) was used at 3 and 5 μg/ml, as a positive control in the portion of the assay performed with activation. The saline test article extract and saline negative control were dosed in 1 ml volumes. The DMSO extracts, DMSO negative controls, and positive controls were dosed in 0.1 ml volumes to minimize solvent toxicity. The S9 fraction plus cofactor pool was applied to the test system at a final concentration of 10%, v/v, where applicable. After dosing, the tubes were incubated at 37 ± 1°C, 5 ± 1% CO_2_ on a shaker (80 rpm). After an exposure period of 4 h, the cells were centrifuged for 5–10 min at 800–1,000 rpm, washed once with approximately 5 ml of growth media, resuspended in a final 20 ml of growth medium, and returned to the incubator. The tubes treated for 24 h remained on the shaker and were washed and resuspended, just before counting on day 2. Since the cells were counted immediately after the treatment period, the 24-h tubes were counted for an extra day to allow for the recovery, growth, and expression of the TK^–/–^ phenotype the same as the short treatments.

##### Expression of Mutants

At approximately 24 h after treatment, each tube was counted and adjusted to 3 × 10^5^ cells/ml in 20 ml of growth medium. The tubes were returned to the incubator for an overnight incubation and adjusted to a final density of 2 × 10^5^ cells/ml in 20 ml of growth medium immediately before cloning. This 2-day incubation period allowed for the recovery, growth, and expression of the TK^–/–^ phenotype.

##### Cloning

From these tubes, an additional 1:100 dilution was made in preparation for viable cell (VC) cloning to determine cloning efficiency (CE). Containing approximately two hundred cells, 100 μl was added to 25 ml aliquots of cloning agar medium, mixed, and poured into a 100-mm Petri plate. Three plates were prepared from each dose tube. To determine mutagenicity, 5 ml from each dose tube (at 2 × 10^5^ cell/ml, for a total of 1 × 10^6^ cells plated) was suspended in 20 ml of selective cloning medium including the restrictive agent TFT on a 100-mm Petri plate. All VC and S (TFT) plates were incubated for 10 to 11 days, after which, the colonies on both VC and S treated plates were counted using an automatic image analyzer, including software for discrimination of colony size.

##### Data analysis

The following values were calculated:

% Relative Cloning Efficiency (% Rel CE) = (avg. VC counts, test group/avg. VC counts, entire neg. control group) × 100.

% Absolute Cloning Efficiency (% Abs CE) = (avg. VC counts test group/total number of cells plated for viability) × 100.

Mutant Frequency (MF) = S counts/VC counts) × (2 × 10^–4^) = mutant × 10^–6^ survivors, where S counts = group sum of mutant colony counts from all selection plates per group, and VC counts = group sum of viable colony counts from all viable cell plates per group.

Global Evaluation Factor (GEF) = mean of the negative MF distribution plus one standard deviation (negative vehicle control MF + 90).

Suspension Growth (SG) 4 h treatment = (day 2 count/day 1 density) × (day 3 count/day 2 adjusted density).

Suspension Growth (SG) 24 h treatment = (day 2 count/day 1 density) × (day 3 count/day 2 adjusted density) × (day 4 count/day 3 adjusted density).

% Relative Suspension Growth (% RSG) = (Suspension growth treated cells/Suspension growth of vehicle control cells) × 100.

% Relative Total Growth (% RTG) = (RSG × Rel CE)/100.

##### Test Evaluation

A test article dose was considered acceptable for evaluation if the Abs CE was 50% or greater, the total viable colonies exceeded approximately 60 colonies, and the %RTG was greater than 10%. A response was considered positive if the test article dosed culture has an induced mutant frequency (IMF) that meets or exceeds the assay’s GEF and is statistically and significantly different from the concurrent negative control. A response was considered negative if the test article dosed culture did not meet the criteria for a positive result. A response was considered equivocal if one of the criteria for a positive result were met, but not all criteria were met.

##### Statistical Analysis

ANOVA (when appropriate, Dunnett’s test) was used to compare the mutant frequency in treated preparations with the concurrent negative control. The difference was considered significant if *p* < 0.05 when comparing the treatment group to the negative control group.

#### Genotoxicity—Mutagenicity Ames Assay (Per ISO 10993-3)

The Ames test evaluates the mutagenic potential of the test article or extracts by measuring the ability to induce DNA mutations at selected loci of several strains of bacteria (e.g., Salmonella, *E. coli*) (Ames et al., 1973; Mortelmans and Zeiger, 2000).

##### Test and Control Article Preparation

Electrodes (*n* = 4; total surface area of 76 cm^2^) were bent, folded, and placed into an extraction vessel at a ratio of 6 cm^2^/1 ml of extraction vehicles, saline, and DMSO for 72 ± 2 h at 50 ± 2°C.

##### Testing

The assay was conducted with four strains of Salmonella typhimurium (TA97a, TA98, TA100, and TA1535) and one strain of Escherichia coli (WP2-uvrA-) in the presence and absence of an exogenous mammalian activation system (S9) (Molecular Toxicology Inc.; Boone, NC, United States). The S9 was mixed with a cofactor pool to contain 5% microsomal enzymes, 5-mM glucose 6-phosphate, 4-mM-nicotine-adenine dinucleotide phosphate, 8-mM MgCl_2_, and 33-mM KCl in a 200-mM phosphate buffer at pH 7.4. Working cultures of the tester strains were prepared from frozen working stocks by transferring the frozen working stock into 40-ml Oxoid nutrient broth and incubated, with shaking, at 37 ± 2°C until an optical density (at 650 nm) of 0.9–1.3 was reached.

For the mutagenicity test, a top agar consisting of 0.6% Difco agar in 0.5% NaCl was melted and a solution of 0.5-mM L-histidine/0.5-mM biotin or 0.5-mM L-tryptophan was added to the melted top agar at a ratio of 10 ml per 100 ml agar. The supplemented agar was aliquoted, 2 ml per tube, and held at 45 ± 2°C. To prepare the top agar for treatment, 0.1 ml of the test article or control, 0.1 ml of the bacteria culture, and 0.5 ml of phosphate-buffered saline were added to the molten agar. The mixture was briefly vortexed and poured onto a room temperature minimal glucose agar plate (1.5% Difco agar, 0.4–2% glucose, in Vogel-Bonner medium E). Metabolic activation was provided by adding 0.5 ml of the S9 mix in place of the PBS. The plates were allowed to harden and then incubated for 48–72 h at 37 ± 2°C. The test article extract was tested in triplicate at one dose level along with appropriate vehicle and positive controls. All treatments were assayed against tester strains TA97a, TA98, TA100, TA1535, and WP2-uvrA- in the presence and absence of metabolic activation.

##### Data Analysis

All plates were counted using an automatic image analysis system. Negative control and test article treated plates were also examined for the presence of a bacterial lawn. For a valid test, all tester strain cultures should exhibit a characteristic number of spontaneous revertants per plate in the negative control treatments. Positive control values must exhibit at least a three-fold increase (FI) over the respective mean negative control value for the strain. FI was defined as (mean test article colony count value)/(mean negative control colony count value). An induced positive result for any strain would be demonstrated by at least a twofold increase in the number of revertant colonies per plate over the negative control values.

#### Hemolysis (Per ISO 10993-4)

##### Test and Control Article Preparation

The test articles were bent, folded, and placed into an extraction vessel at a ratio of 6 cm^2^/1 ml of calcium and magnesium-free phosphate-buffered saline (CMF-PBS), for 72 h at 50°C. The negative control was HDPE extracted in CMF-PBD. The positive control was Sterile Water for Injection.

##### Animals

Whole blood was collected from three adult female New Zealand White rabbits.

##### Preparation

Samples were pooled, diluted, and added to polystyrene tubes with CMF-PBS test article extract. Negative controls, positive controls, and blanks were prepared similarly. Following incubation for at least 3 h at 37°C, the samples were centrifuged, and each supernatant was collected. The supernatant was mixed with Drabkin’s reagent, and the resulting solution was analyzed using a spectrophotometer at a wavelength of 540 nm (Lin et al., 2020).

##### Data Analysis

The mean blank corrected percentage (%) hemolysis (BCH) was calculated by averaging the blank corrected % hemolysis values of the triplicate test samples. In the event the BCH resulted in a value less than zero, the value was reported as 0.00. The standard deviation (SD) for the replicates was determined. An average hemolytic index of the triplicate test samples was also calculated as follows: Hemolytic Index = Mean BCH (Test Article)- Mean BCH (Negative Control). A hemolytic index value between 0 and 2 is classified as non-hemolysis.

#### Sensitization (Per ISO 10993-10)

This test is designed to evaluate the allergenic potential or sensitizing capacity of a test article (Magnusson and Kligman, 1969; Schlede and Eppler, 1995). The test consists of two phases, induction and challenge. The induction phase includes exposing a test group of animals twice to the test material; first by intradermal injection followed by topical application 7 days later. During Induction A, the test animals are exposed intradermally to the test material, along with an adjuvant to enhance the immune reaction of the guinea pig. During Induction B, the topical induction, the test group is exposed to the test article for 48 h, occluded.

##### Test and Control Article Preparation

Electrodes (*n* = 18, total surface area of 342 cm^2^) were bent, folded, and placed into an extraction vessel at a ratio of 6 cm^2^/1 ml of extraction vehicles for 72 ± 2 h at 50 ± 2°C. The vehicles were 0.9% NS or SO. The extracted media was used for intradermal injection.

##### Animals

Adult female albino guinea pigs (Cavia porcellus; 300–500 g; *n* = 51), were obtained from Robinson Services. Animals were acclimated for a minimum of 5 days before testing and had food and water *ad libitum* through the study. Animals were divided into the following groups: (1) test group NS (*n* = 11; test article dissolved in NS), (2) test group SO (*n* = 11; test article dissolved in SO), (3, 4) negative control groups (*n* = 6 for NS and *n* = 6 for SO), (5) positive control group (*n* = 11; dinitrochlorobenzene, DNCB), and (6) vehicle group for the positive control group (*n* = 6; a vehicle for DNCB). Before the induction phases, an approximate 5 cm × 7 cm area over the shoulder region was shaved. Before the challenge phase, an approximate 4 cm × 4 cm area of the right and the left flank was shaved.

##### First Induction/Intradermal Injection

Each animal in the test or negative control group received the following 6 injections listed in Table 1, in a volume of 0.1 ml in the shoulder region (3 injections on each the right and left side, within the boundaries of a 2 cm × 4 cm area). The Freund’s complete adjuvant (FCA) was used to enhance the potential of weak sensitizing agents, and thereby maximize the response. Animals in the positive control and vehicle groups received 0.3% dinitrochlorobenzene (DNCB) in ethanol and ethanol, respectively.

**TABLE 1:** Intradermal injections for guinea pig sensitization test.

Sites	Injection content	Ratio (v/v)
**Test group (*n* = 22 animals; 11 for NS as a vehicle and 11 for SO as a vehicle)**		
Site 1 L, R	FCA + 0.9% sterile saline	1:1
Site 2 L, R	Test extract (NS or SO)	NA
Site 3 L, R	FCA + 0.9% sterile saline (1:1) + test extract (NS or SO)	1:1
**Negative control group (*n* = 12 animals; 6 for NS as a vehicle and 6 for SO as a vehicle)**		
Site 1 L, R	FCA + 0.9% sterile saline	1:1
Site 2 L, R	Control vehicle (NS or SO)	NA
Site 3 L, R	FCA + 0.9% sterile saline (1:1) + control vehicle (NS or SO)	1:1

*NS, normal saline; SO, sesame oil; L, left; R, right; FCA, Freund’s complete adjuvant.*

##### Second Induction/Topical Application

Six days after the intradermal injections, the injection site areas were clipped free of fur and treated with 0.5 ml of 10% (w/w) Sodium Lauryl Sulfate (SLS) prepared by mixing solid SLS with mineral oil. On Day 7, the test article extract (0.3 ml) was applied to a 2 cm × 4 cm piece of filter paper to saturation, and then, applied to the treatment site. The patch was secured to the site with non-permeable tape. The trunk of each animal was wrapped with an elastic bandage and hypoallergenic tape. The negative control animals received a similar patch to the control vehicles. The preparations were removed after 48 ± 2 h.

##### Challenge Patch/Topical Application

Fourteen days after completion of the topical induction phase, the challenge procedure was initiated. A 2 cm × 2 cm filter paper patch was saturated with 0.3 ml of test article extract or control vehicle. In the test group animals, the filter papers with the test article and vehicle were placed on the left and right flank areas of each animal, respectively. The negative control group animals were challenged identically with similarly prepared patches. The positive control and vehicle groups were challenged identically with patches containing 0.15% DNCB in acetone and acetone, respectively. The trunk of each animal was wrapped with an elastic bandage and hypoallergenic tape. The preparations were removed after 48 ± 2 h.

##### Test Evaluation

The challenge sites were observed at 24 ± 2 and 48 ± 2 h after patch removal for irritation and sensitization reaction, as indicated by erythema and edema, using a grading scale for skin reactions: 0 = no visible change – no erythema and edema, 1 = discrete or patchy erythema, 2 = moderate and confluent erythema, 3 = intense erythema and/or swelling, per ISO 10993-10, using the Magnusson and Kligman scale (Magnusson and Kligman, 1969; Schlede and Eppler, 1995). Any other adverse changes at the skin sites were recorded and reported. Grades of 1 or greater in the test group generally indicate sensitization and provided grades less than 1 in the control group.

#### Irritation (ISO 10993-10)

##### Test and Control Article Preparation

Electrodes (*n* = 4, total surface area 76 cm^2^) were bent, folded, and placed into an extraction vessel at a ratio of 6 cm^2^/1 ml of extraction vehicles for 72 ± 2 h at 50 ± 2°C. The vehicles were 0.9% NS or SO.

##### Animals

Adult female nulliparous and non-pregnant albino rabbits (*Oryctolagus cuniculus*, New Zealand White strain, 2 kg; *n* = 6), were obtained from Robinson Services. Animals were acclimated for a minimum of 5 days before testing and had food and water *ad libitum* through the study. At the end of the study, all animals were euthanized with sodium pentobarbital.

##### Testing

**FIGURE 1:**
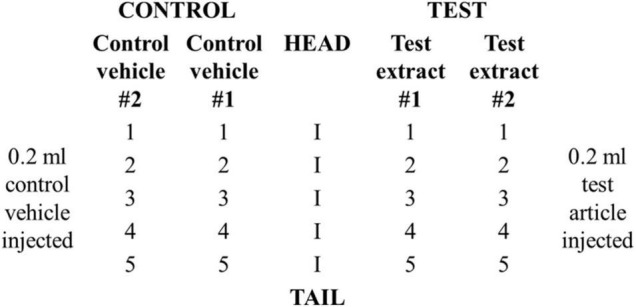
Schematic of injection sites for rabbit irritation test.

Animals were divided into a test (*n* = 3) and a positive control group (*n* = 3). Each rabbit in the test group received a total of 20 intracutaneous injections consisting of 0.2 ml of test article dissolved in vehicle #1, NS, test article dissolved in vehicle # 2, SO, vehicle #1, NS, and vehicle #2, SO. Two sets of five injections were administered on the right and left sides of the vertebral column (parallel and distant, ∼2 cm apart) according to the scheme shown in Figure 1. Animals in the positive control group received injections of 0.15% SLS (dissolved in 0.9% NS) as the test solution and 0.9% NS as the control vehicle.

##### Test Evaluation

Injection sites were evaluated for gross evidence of erythema and edema at 24 ± 2, 48 ± 2, and 72 ± 2 h using the following grading system: 0 = no erythema, edema; 1 = very slight erythema, edema; 2 = well-defined erythema, edema (edges of area well-defined by definite raising), 3 = moderate erythema, edema (raised ∼1 mm); and 4 = severe erythema (beet redness) to eschar formation preventing grading of erythema, severe edema (raised > 1 mm and extending beyond exposure area). For each, erythema and edema, all grades obtained at each time point were totaled separately for each test sample or control for each animal. To calculate the score of a test sample or control on each animal, each of the totals was divided by 15 (three scoring time points × five test or control sample injection sites). To determine the overall mean score for each test sample and each corresponding control, the scores were added for the three animals and divided by three. The final test sample score was obtained by subtracting the score of the control from the test sample score. If the difference between the average scores for the extract of the test article and the vehicle control is less than or equal to 1, the test article is considered to have met the requirements of the test.

#### Acute Systemic Toxicity (per ISO 10993-11)

##### Test and Control Article Preparation

Electrodes (*n* = 7, total surface area 133 cm^2^) were bent, folded, and placed into an extraction vessel at a ratio of 6 cm^2^/1 ml of extraction vehicles for 72 ± 2 h at 50 ± 2°C. The vehicles were 0.9% NS or SO.

##### Animals

Adult female nulliparous and non-pregnant albino Swiss mice (Mus musculus, ND4 *n* = 10), were obtained from ENVIGO. Animals were acclimated for a minimum of 5 days before testing and had food and water *ad libitum* through the study. All animals were euthanized by CO_2_ asphyxiation at the end of the study.

##### Testing

Groups of five animals were injected with either the test article extract or the corresponding control vehicle (NS and SO) at a dose of 50 ml/kg via two routes of administration, intravenous (i.v.; infusion rate ∼0.1 ml/sec) and intraperitoneal (IP).

##### Test Evaluation

Animals were evaluated for mortality, signs of pharmacological and/or toxicological effects, and weight loss incidence at 4 ± 0.75, 24 ± 2, 48 ± 2, and 72 ± 2 h post-injection. The test is considered negative if none of the animals injected with the test article show a significantly greater biological reaction than the animals treated with the control vehicle. Death in two or more mice or other toxic signs, such as convulsions, prostration, or bodyweight loss greater than 10% in three or more mice, is interpreted as significant biological reactions.

#### Pyrogenicity (Per ISO 10993-11)

##### Test and Control Article Preparation

Electrodes (*n* = 48; total surface area 912 cm^2^) were bent, folded, and placed into an extraction vessel at a ratio of 6 cm^2^/1 ml of extraction vehicle for 72 ± 2 h at 50 ± 2°C. The vehicle was 0.9% NS.

##### Animals

Adult female nulliparous and non-pregnant albino rabbits (*Oryctolagus cuniculus*, New Zealand White strain, 2 kg; *n* = 6), were obtained from Robinson Services. Animals were acclimated for a minimum of 5 days before testing and had food and water *ad libitum* through the study. Before using the rabbits for the first time in a pyrogen test, they were conditioned to the physical requirements of the procedure with a sham test. This included all the steps and documentation as directed in the pyrogen test, except injection. All animals were euthanized with sodium pentobarbital at the end of the study.

##### Testing

The test was performed in a separate area designated for pyrogen testing, under environmental conditions similar to those under which rabbits are housed, and free from any disturbances that might excite them. The rabbits were restrained with light-fitting neck stocks that allowed the animals to assume a natural resting position with a rectal probe in place for the course of the study (3 h) and were not offered food or water during the testing period. Baseline temperature was measured in a window of 30 min before injection. The test article extract was warmed to 37 ± 2°C and injected slowly and steadily in the ear vein at 10 ml/kg. Each injection was completed within 10 min of initiation. The temperature was measured at 30-min intervals between 1 and 3 h post-injection. An individual temperature increase of 0.5°C, or a cumulative temperature increase of 3.3°C, is considered a positive indication of pyrogenicity.

#### Implantation (Per ISO 10993-6)

##### Animals

This study was conducted in compliance with the FDA GLP Regulations, 21 CFR Part 58, and ISO 10993-6:2016 and 10993-12:2012 guidelines. Adult sheep (Polypay breed, 73.37 ± 7.07 kg, *n* = 9 total, 7 males, 2 females) were obtained from Purdue University (West Lafayette, Indiana, United States). Animals were implanted with either the test article or a control material, using fluoroscopy for guidance. The test article was a thin film electrode (1 × 4 strip) with dimensions 8 × 35 × 0.08 mm. The control material consisted of USP HDPE and was 8 × 8 × 1 mm. Ten test articles (5 animals, two implants per animal) and 12 control articles (4 animals, three implants per animal) were placed in the subdural space over the right cerebral hemisphere for 28 days, determined by the intended clinical exposure period. Throughout the length of the study, animals were monitored for changes in skin and fur, eyes and mucous membranes, respiratory, circulatory, autonomic, and central nervous system, and somatomotor activity and behavior patterns. Blood samples were collected for standard hematology and serum chemistry analysis before implantation and at the terminal experiment.

##### Tissue Collection and Histology

Animals were euthanized at the end of the survival period and necropsy was performed for target tissue procurement. The implant sites were explanted, fixed in 10% Neutral buffered formalin, and embedded in paraffin. The tissue was sectioned at 5 μm and stained with Hematoxylin and Eosin (H&E). All slides were visualized under a light microscope and semi-quantitatively scored by a certified pathologist. The tissue was assessed for accumulation of immune system cells (polymorphonuclear cells, lymphocytes, plasma cells, neutrophils, lymphocytes, macrophages/gitter cells, and multinucleated giant cells), neovascularization, fibrosis, and astrocytosis/fatty infiltration, according to the criteria listed in ISO 10993-6:2016.

## Results

### Cytotoxicity

The effects of leachable and/or diffusible substances from the test article on the morphology of mammalian L929 cell cultures were examined after incubation with the test article extract and controls for 24, 48, and 72 h. The cell layer reactivity was scored on a scale of 0 to 4 for abnormal cell morphology and cellular degeneration, which included lysis, crenation, plaques, and excessive rounding of cells. The test article and the negative control (high-density polyethylene, HDPE) scored “0” at all-time points (Table 2). In contrast, the positive control (polyurethane film containing 0.1% zinc diethyldithiocarbamate, ZDEC), scored 4 at each time point (Table 2). Based on these results, the test article is considered non-cytotoxic under the conditions of this test.

**TABLE 2:** Cytotoxicity testing results.

Test article and controls	Cytotoxicity score		
	24 h	48 h	72 h
Test article	0/0/0	0/0/0	0/0/0
Positive control	4/4/4	4/4/4	4/4/4
Negative control	0/0/0	0/0/0	0/0/0
Cell control	0/0/0	0/0/0	0/0/0

*Semiquantitative analysis of cell layer reactivity after 24, 48, and 72 h exposure to extracts from the test article (thin-film electrodes), positive control (polyurethane film containing 0.1% ZDEC), and negative control (HDPE).*

### Genotoxicity—Mouse Lymphoma Assay

The test article extract was in contact with the test system for 4 h in the presence and absence of metabolic activation and 24 h in the absence of metabolic activation. Cloning efficiency, relative total growth (RTG), and mutant frequency (MF) are presented in Tables 3, 4. Neither test article extract (either with or without metabolic activation or the extended treatment time) induced appreciable differences in cell density throughout the expression and recovery period as compared to the concurrent negative control. The absolute cloning efficiencies of preparations treated with the extracts in the presence or absence of metabolic activation were within acceptable ranges (device saline extract: 72%; device saline extract + S9: 70%; device saline extract 24 h: 61%; device DMSO extract: 65%; device DMSO extract + S9: 77%; device DMSO extract 24 h: 66%) (Table 3). No test article treatment induced substantial changes in% RTG, indicating no notable levels of cytotoxicity.

**TABLE 3:** Cloning efficiencies (CE) and relative total growth (RTG).

Parameter	Group Abs CE			Group Rel CE			Average RTG		
**Treatment**	**A**	**B**	**C**	**A**	**B**	**C**	**A**	**B**	**C**
Device extract saline (%)	70	72	61	96	110	92	109	124	87
Device extract DMSO (%)	77	65	66	105	99	92	75	104	89
Vehicle control saline (%)	72	65	67	NA	NA	NA	100	100	100
Vehicle control DMSO (%)	73	66	72	NA	NA	NA	100	99	99
A: 5 μg/ml CP (%) B: 15 μg/ml MMS (%)	45	44	NA	63	67	NA	60	59	NA
A: 3 μg/mL CP (%) B, C: 10 μg/ml MMS (%)	55	56	49	76	86	74	102	86	48

*Treatment A is 4 h with metabolic activation, treatment B is 4 h without metabolic activation, and treatment C is 24 h without metabolic activation. NA, not applicable.*

**TABLE 4:** Mutant frequency (×10^–6^).

Treatment	Device extract saline	Device extract DMSO	Vehicle control saline	Vehicle control DMSO	Low dose positive control	High dose positive control
Without S9	76.2	57.8	46.5	50.0	369.6	510.0
Without S9, 24 h	55.1	54.7	58.6	47.6	355.3	NA
With S9	73.0	57.1	52.2	52.6	211.9	512.6

The mutant frequencies of all preparations treated with the test article extracts were not different from those treated with the concurrent negative control (Table 4). Actual colony counts did not show increases in absolute numbers of colonies present in any test article extract-treated preparation (data not shown). Additionally, none of the test article-treated groups showed biologically significant increases in mutant frequency as compared to the concurrent negative control under any condition.

The mutant frequencies and cloning efficiencies of preparations treated with the test articles were within the limits defined for a negative response. Accordingly, the test article is considered to be both non-mutagenic and non-clastogenic in this test system.

### Genotoxicity—Mutagenicity Ames Assay

The test article did not induce substantial increases in reversion rates of the type that are associated with mutagenesis (Table 5). No substantial test article toxicity was noted that may have interfered with the ability of the test system to detect mutagens. As none of the tester strains showed an increase in reversion rates when treated with the test article, the test article is determined to not have caused an increase in point mutations, exchanges, or deletions. Based on the criteria and conditions of the study protocol, the test article is considered non-mutagenic.

**TABLE 5:** Colony count data.

		With S9 activation					Without S9 activation				
System		Device extract saline	Saline control	Device extract DMSO	DMSO control	Positive control	Device extract saline	Saline control	Device extract DMSO	DMSO control	Positive control
TA97a	**Mean ± SD**	133.7 ± 10.0	124.7 ± 7.0	125.0 ± 19.1	108.7 ± 7.5	2,003.7 ± 93.3	107.3 ± 19.3	112.0 ± 12.2	103.3 ± 5.0	98.7 ± 6.5	1,158.0 ± 28.0
	**FI**	1.1	NA	1.2	NA	16.1	1.0	NA	1.0	NA	10.3
TA98	**Mean ± SD**	35.0 ± 2.6	36.0 ± 3.5	29.7 ± 8.1	33.7 ± 2.5	3,016.0 ± 23.4	30.3 ± 6.7	22.0 ± 7.0	20.7 ± 4.2	29.3 ± 3.2	1,298.3 ± 25.8
	**FI**	1.0	NA	0.9	NA	83.8	1.4	NA	0.7	NA	59.0
TA100	**Mean ± SD**	133.0 ± 26.1	118.7 ± 6.1	123.7 ± 6.8	133.3 ± 12.4	3,484.7 ± 80.9	136.7 ± 10.8	125.3 ± 4.0	121.7 ± 9.6	109.7 ± 4.9	992.0 ± 13.1
	**FI**	1.1	NA	0.9	NA	29.4	1.1	NA	1.1	NA	7.9
TA1535	**Mean ± SD**	13.0 ± 4.4	14.3 ± 2.9	13.0 ± 4.6	11.3 ± 3.5	226.3 ± 16.0	12.0 ± 1.7	13.0 ± 3.6	13.7 ± 3.5	13.7 ± 3.1	968.3 ± 51.0
	**FI**	0.9	NA	1.1	NA	15.8	0.9	NA	1.0	NA	74.5
WP2-uvrA-	**Mean ± SD**	36.3 ± 7.0	40.3 ± 13.4	52.7 ± 5.1	40.3 ± 1.5	148.7 ± 15.9	42.7 ± 8.1	43.3 ± 5.7	31.0 ± 1.0	38.0 ± 9.8	416.7 ± 40.5
	**FI**	0.9	NA	1.3	NA	3.7	1.0	NA	0.8	NA	9.6

*Data (mean and SD) for each system represent an average of three repetitions. NA, not applicable; SD, standard deviation of the group; FI, fold increase, compares the test group with the concurrent negative control group and it is defined as FI = (mean test article colony count value)/(mean negative control colony count value).*

### Hemolysis

The hemolytic index for the test article extract was 0.3% (Table 6), considered non-hemolytic.

**TABLE 6:** Hemolysis.

Sample	Mean	SD	Mean Hg concentration (mg/ml)	Hemolytic index
Test article	0.33	0.0	0.01	0.3
Negative control	0.05	0.0	0.01	n/a
Positive control	101.46	0.6	1.30	n/a
Blanks	0.65*	0.1	n/a	n/a

Measurements of the hemolytic index in red blood cells exposed extracts from the test article (thin-film electrodes), positive control (sterile water for injection), and negative control (HDPE). *Replicate/Mean% Hemolysis; n/a, not applicable.

### Sensitization

No animal (*n* = 34 guinea pigs) showed abnormal clinical signs during the test period. The challenge sites were assessed for irritation and sensitization reaction, as indicated by erythema and edema. At both 24 and 48 h after the challenge, none of the test animals (*n* = 11) challenged with the test article extracts were observed with a sensitization response greater than “0” (i.e., no edema and erythema) (raw data shown in Supplementary Table 1). None of the negative-control animals challenged with the control vehicles (*n* = 6) were observed with a sensitization response greater than “0” (raw data shown in Supplementary Table 1). At both 24 and 48 h after the challenge, all animals in the positive control group (*n* = 11) were observed with discrete or patchy erythema (scores of “1| “) or moderate and confluent erythema (scores of “2”) at the challenge sites, indicating a 100% sensitization response (raw data shown in Supplementary Table 2). By contrast, none of the animals in the vehicle group (*n* = 6) exhibited erythema (scores of “0”) at the challenge sites (raw data shown in Supplementary Table 2). These results indicate that under the conditions of this protocol, the test article did not elicit a sensitization response.

### Irritation

There were no abnormal clinical signs during the 24, 48, and 72 h observation periods in any of the animals (*n* = 6 rabbits). Evaluation of the local irritation reaction (inflammation, redness, swelling, heat, and/or pain) was performed by semiquantitative scoring of edema and erythema. The score for the positive control group was 2.3 (raw data shown in Supplementary Table 3). The scores for the test article dissolved in NS or SO were 0 and 0.1, respectively (raw data shown in Supplementary Tables 4, 5), indicative of no irritation.

### Acute Systemic Toxicity

At the end of the test period, at 72 h, all animals in the control and test groups (*n* = 5 Swiss mice per group) were alive, and none exhibited abnormal clinical signs indicative of toxicity and none lost weight over 10%. These findings indicate no acute systemic toxicity.

### Pyrogenicity

No animal had a baseline temperature above 39.8°C or less than 38.5°C. The maximum temperature rise for the three test rabbits was 0.2°C. The animal body weights, dose volumes, baseline temperatures, and test temperature results are reported in Supplementary Table 6. These results indicate that the test article is non-pyrogenic.

### Implantation

**FIGURE 2:**
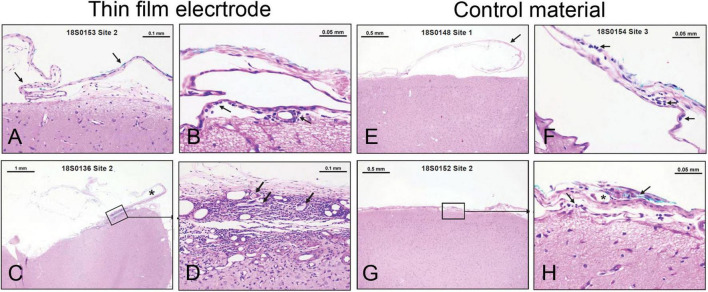
Minimal or no reaction to electrode implant for 28 days in sheep brain. **(A–D)** Examples from thin-film electrode sites. **(A)** No tissue reaction. **(B)** Rare macrophages (arrow) present in the arachnoid-pia region. **(C)** Partially disrupted fibrous capsule (asterisk-implant site). **(D)** Higher magnification from panel **C** illustrating mixed cellular infiltrates, foreign materials (cotton fibers— arrows), fibrosis, and neovascularization. **(E–H)** Examples from control material (USP HDPE) sites. **(E)** No tissue reaction. **(F)** Rare lymphocytes and macrophages (arrows) are present in the arachnoid membrane. **(G,H)** A focus of cellular infiltrates (arrows) at the implant site centered on a polarizable foreign material (cotton fibers—asterisk) within the arachnoid mater.

The duration of the implantation was 28 days, which covers the average use of these electrodes in the Epilepsy Monitoring Unit (EMU) [8.12 ± 3.49 days (mean ± SD) with a range of 2–29 days (Mullin et al., 2016; Punia et al., 2018; Sacino et al., 2019; Kim et al., 2020, 2021]. The brain and associated arachnoid-pia in contact with each implant were evaluated *via* histopathology for accumulation of immune system cells (polymorphonuclear cells, lymphocytes, plasma cells, neutrophils, lymphocytes, macrophages/gitter cells, and multinucleated giant cells), necrosis, neovascularization, fibrosis, and astrocytosis/fatty infiltration. Semiquantitative scores for each site are presented in Supplementary Tables 7, 8. The group average scores were 3.4 for the test article and 1.7 for the control article (USP HDPE). The calculated test article relative score for this study was 1.7, and the resulting test article characterization was interpreted to be a reactivity grade of minimal or no reaction. Representative photomicrographs demonstrating test article and control article implant site characteristics are shown in Figure 2.

## Discussion

This study tested the biocompatibility of thin-film subdural electrodes made of polyimide as a substrate with 3-mm diameter platinum contacts. All tests were performed under GLP conditions and at the standards specified by ISO 10993. The battery of tests included *in vitro* cytotoxicity, genotoxicity and hemolysis and *in vivo* sensitization, irritation, acute systemic toxicity, pyrogenicity, and a 28-day brain implantation. The result indicated that the electrodes were non-cytotoxic, non-mutagenic, non-clastogenic, non-hemolytic, non-pyrogenic, exhibited no systemic toxicity, and elicited no or minimal tissue immunological reaction. These results demonstrated device safety and led to the first FDA clearance of a thin-film electrode technology for a subdural implant for monitoring and recording brain activity for up to 30 days. The electrodes have been in clinical use since November 2020.

Several materials are currently used for electrodes intended to be implanted in the brain. Traditional subdural electrodes, which have been on the market for many years, are made of a silicon substrate with platinum or platinum-iridium contacts. Platinum is commonly used in electrodes intended for neural recording and stimulation because it resists corrosion, has demonstrated good biocompatibility in the brain, and it is amenable to electrode fabrication processes. These properties ensure the long-term reliability of electrodes for chronic recordings and stimulation (Negi et al., 2010; Weremfo et al., 2015; Shokoueinejad et al., 2019). Studies have shown that tissue reaction to implanted electrodes depends on the electrode properties, including materials, size, shape, stiffness, surface, and others (Carnicer-Lombarte et al., 2021). Silicone-based electrodes do not properly conform to the brain surface (Tong et al., 2020). Electrodes made of soft materials, sufficiently thin and more flexible are preferable. Polyimide is a high-performance polymer increasingly considered for neural implants due to its good thermal stability (>500°C), biocompatibility, mechanical toughness, chemical resistance, and long term stability when implanted (Richardson et al., 1993; Geddes and Roeder, 2003; Rubehn and Stieglitz, 2010; Hassler et al., 2011; McKeen, 2014a,b; Constantin et al., 2019; He et al., 2020; Vomero et al., 2020, 2022; Schander et al., 2021). Polyimide as a substrate, in combination with other metals including platinum, has been used in other neural or retinal implants. Cytotoxicity of these devices or extracts was evaluated in mouse fibroblasts (Richardson et al., 1993; Sun et al., 2009; Bae et al., 2012; Park et al., 2014; Lin et al., 2020), rat neurons (Lacour et al., 2008), human (Seo et al., 2004) and rat retinal epithelial cells (Julien et al., 2011), and human endothelial cells (Starr et al., 2016), and have shown to be negligible. Similar to these studies, our results showed no abnormal cell morphology, and no cellular degeneration, establishing that these thin-film electrodes are non-cytotoxic. Polyimide does not exhibit hemolytic properties. When tested by itself, polyimide’s hemolytic index was intermediate between the values observed for Teflon and Silastic controls (Richardson et al., 1993). When tested in neural prosthesis made of titanium and platinum composites deposited in a silicon wafer and encapsulated in polyimide, the hemolytic index was also < 2, considered non-hemolytic (Lin et al., 2020). These neural prostheses have also shown no sensitization or irritation, similar to our results when tested in the same model and using similar methods as used here (Lin et al., 2020). Pyrogenicity is a very important factor in ensuring the safety of new medical devices. Although there are many causes of medical device-induced pyrogenicity, one source is the material mediated pyrogenicity, which refers to any exogenous and non-biological substance that can cause a febrile response. These are thought to leach out from the device materials or surfaces (review Borton and Coleman, 2018). Our results indicate that the thin-film subdural electrodes investigated here are not pyrogenic. Mutations can lead to cancer, therefore, genotoxicity is a critical part of biocompatibility. Our results showed that the mutant frequencies and cloning efficiencies of cells treated with the test article were within the limits defined for a negative response, demonstrating that the test article is non-mutagenic and non-clastogenic in this test system.

Implanted materials have an impact on the local tissue and its environment. Tissue’s immunological reaction to implanted electrodes is a complex process characterized by a multitude of biochemical and immunological reactions occurring in a timely fashion at the electrode-tissue interface (Anderson, 2001; Gulino et al., 2019; Sung et al., 2020). The early (hour to weeks) response is characterized by an acute inflammatory response involving the accumulation of immune system cells (macrophages, monocytes), blood-borne macrophages, and edema, followed by activation and migration of microglial cells (Anderson, 2001; Polikov et al., 2005; Gulino et al., 2019; He et al., 2020; Sung et al., 2020). Indeed, histopathological findings from tissue resected from patients with epilepsy, who had invasive EEG monitoring with subdural electrodes and/or depth electrodes for a median of 7 days, have shown chronic inflammation with an accumulation of lymphocytes and macrophages, contusion or acute/subacute infarct, acute inflammation, acute hemorrhage, edema, and necrotizing vasculitis (Fong et al., 2012).

Our study in sheep revealed minimal tissue reaction (Figure 2 and Supplementary Tables 7, 8), which was similar to that elicited by the control material (HDPE). Among the reasons for lack or minimal tissue reaction might be the physical properties of the electrodes, such as thickness and reduced weight. Thickness is an important factor that can contribute to heightened tissue reaction because of the mechanical pressure exerted by the electrodes on the brain, when being compressed between the brain and skull plate. It is conceivable that the thicker the electrode, the higher the pressure. Pressure on the cortical tissue compresses the cortical veins and interrupts cerebral blood flow, resulting in vasogenic edema and blood and fluid accumulation in the subarachnoid space, which can lead to an increase in intracranial pressure, bleeding (hematoma), and microinfarcts (Araki et al., 2006; Mocco et al., 2006; Van Gompel et al., 2008; Fong et al., 2012). A previous report on silicone electrodes has directly linked the lack of reliable conformability to brain surface to an increased number of complications (Tong et al., 2020). The study analyzed complication rates in patients implanted with subdural electrodes at one center over 14 years and found that electrodes do *“not reliably conform to the convex surface of the cortex*,” which could promote fluid/blood accumulation in the spaces between electrodes and dura and between dura and skull. This accumulation can cause a “mass effect,” putting pressure on the brain, which has been linked to post-operative complications, including increased intracranial pressure, intracranial hemorrhage, infections, and neurologic compromise. Changes to improve electrode conformability, by making incisions in the plastic sheets and decreasing the effects of thickness/bulkiness and by using dural expansion, dramatically reduced post-operative complications (Tong et al., 2020). Together, the physical properties (e.g., thickness, reduced weight) of these new thin-film polyimide subdural electrodes, coupled with good biocompatibility, may have the potential to improve clinical outcomes.

## Conclusion

This study presented a battery of biocompatibility tests designed to assess the device safety of new thin-film polyimide electrodes for a subdural implant. The results demonstrated that the electrodes are non-cytotoxic, non-mutagenic, non-clastogenic, non-hemolytic, non-pyrogenic, exhibited no systemic toxicity, and elicited no or minimal tissue immunological reaction. These properties may help improve clinical outcomes, e.g., by reducing the complications associated with prolonged brain activity monitoring in patients with DRE. These electrodes were the first thin-film electrodes to be cleared by the FDA and have been in clinical use since November 2020.

## Data Availability Statement

The original contributions presented in the study are included in the article/Supplementary Material, further inquiries can be directed to the corresponding author/s.

## Ethics Statement

The animal study was reviewed and approved by the Institutional Animal Care and Use Committee (IACUC) at American Preclinical Services (APS, now part of NAMSA; Minneapolis, MN, United States), IACUC at WuXI AppTec laboratories (St Paul, MN, United States), and IACUC at NAMSA (Northwood, OH, United States).

## Author Contributions

AK wrote the manuscript. DK, SM, DR, MC, CD-B, and AK designed and supervised the execution of the experiments. DR obtained funding for the study. All authors provided scientific input for the manuscript and approved its final version.

## Conflict of Interest

This study received funding from NeuroOne Medical Technologies Corporation. All authors are employees of NeuroOne Medical Technologies Corporation. The funder was not involved in the study design, collection, analysis, interpretation of data, the writing of this article or the decision to submit it for publication. All authors declare no other competing interests.

## Publisher’s Note

All claims expressed in this article are solely those of the authors and do not necessarily represent those of their affiliated organizations, or those of the publisher, the editors and the reviewers. Any product that may be evaluated in this article, or claim that may be made by its manufacturer, is not guaranteed or endorsed by the publisher.
