# Supplementary material for: First Food and Drug Administration Cleared Thin-Film Electrode for Intracranial Stimulation, Recording, and Monitoring of Brain Activity—Part 1: Biocompatibility Testing
Source: Front Neurosci. 2022 Apr 29;16:876877. doi: 10.3389/fnins.2022.876877 (PMC9100917; doi:10.3389/fnins.2022.876877)
Supplement: Supplementary file 1 [file Table_1.docx]

Supplementary Material

**Manuscript: First FDA cleared thin film electrode for intracranial stimulation, recording and monitoring of brain activity - Part 1: biocompatibility testing**

**Supplemental Table 1. Sensitization scores for the test and control vehicles groups.** Challenge sites were evaluated for irritation and sensitization skin reaction as indicated by erythema and edema. Daily challenge observation scores were recorded 24 ± 2 and 48 ± 2 h after patch removal using a grading scale for skin reactions: 0 = no visible change, no erythema and edema, 1 = discrete or patchy erythema, 2 = moderate and confluent erythema, 3 = intense erythema and/or swelling, following the Magnusson and Kligman scale (Magnusson and Kligman, 1969; Schlede and Eppler, 1995).

| **A. Normal Saline (NS)** | | | | | |
| --- | --- | --- | --- | --- | --- |
| **Animal #** | **24 Hour Scores** | | **48 Hour Scores** | | **Results**  **(+) or (-)** |
|  | **Test Group** | | | |  |
|  | **Control Vehicle** | **Test Extract** | **Control Vehicle** | **Test Extract** |  |
| 0340 | 0 | 0 | 0 | 0 | - |
| 0341 | 0 | 0 | 0 | 0 | - |
| 0315 | 0 | 0 | 0 | 0 | - |
| 0343 | 0 | 0 | 0 | 0 | - |
| 0344 | 0 | 0 | 0 | 0 | - |
| 0333 | 0 | 0 | 0 | 0 | - |
| 0279 | 0 | 0 | 0 | 0 | - |
| 0347 | 0 | 0 | 0 | 0 | - |
| 0297 | 0 | 0 | 0 | 0 | - |
| 0349 | 0 | 0 | 0 | 0 | - |
| 0350 | 0 | 0 | 0 | 0 | - |
| **Animal #** | **Negative Control Group** | | | | **Results (+) or (-)** |
|  | **Control Vehicle** | **Test Extract** | **Control Vehicle** | **Test Extract** |  |
| 0334 | 0 | 0 | 0 | 0 | - |
| 0335 | 0 | 0 | 0 | 0 | - |
| 0336 | 0 | 0 | 0 | 0 | - |
| 0337 | 0 | 0 | 0 | 0 | - |
| 0338 | 0 | 0 | 0 | 0 | - |
| 0339 | 0 | 0 | 0 | 0 | - |

| **B. Sesame Oil (SO)** | | | | | |
| --- | --- | --- | --- | --- | --- |
| **Animal #** | **24 Hour Scores** | | **48 Hour Scores** | | **Results (+) or (-)** |
|  | **Test Group** | | | |  |
|  | **Control Vehicle** | **Test Extract** | **Control Vehicle** | **Test Extract** |  |
| 0358 | 0 | 0 | 0 | 0 | - |
| 0359 | 0 | 0 | 0 | 0 | - |
| 0360 | 0 | 0 | 0 | 0 | - |
| 0361 | 0 | 0 | 0 | 0 | - |
| 0362 | 0 | 0 | 0 | 0 | - |
| 0363 | 0 | 0 | 0 | 0 | - |
| 0364 | 0 | 0 | 0 | 0 | - |
| 0365 | 0 | 0 | 0 | 0 | - |
| 0366 | 0 | 0 | 0 | 0 | - |
| 0367 | 0 | 0 | 0 | 0 | - |
| 0368 | 0 | 0 | 0 | 0 | - |
| **Animal #** | **Negative Control Group** | | | | **Results (+) or (-)** |
|  | **Control Vehicle** | **Test Extract** | **Control Vehicle** | **Test Extract** |  |
| 0352 | 0 | 0 | 0 | 0 | - |
| 0353 | 0 | 0 | 0 | 0 | - |
| 0354 | 0 | 0 | 0 | 0 | - |
| 0355 | 0 | 0 | 0 | 0 | - |
| 0351 | 0 | 0 | 0 | 0 | - |
| 0357 | 0 | 0 | 0 | 0 | - |

**Supplemental Table 2. Sensitization scores for the positive control group.** Challenge sites were evaluated for irritation and sensitization skin reaction as indicated by erythema and edema. Daily challenge observation scores were recorded 24 ± 2 and 48 ± 2 h after patch removal using a grading scale for skin reactions: 0 = no visible change, no erythema and edema, 1 = discrete or patchy erythema, 2 = moderate and confluent erythema, 3 = intense erythema and/or swelling, according to ISO 10993-10 guidelines. DNCB = Dinitrochlorobenzene

| **24 Hour Scores** | | | **48 Hour Scores** | | | **Results (+) or (-)** |
| --- | --- | --- | --- | --- | --- | --- |
| **Positive control group** | | | | | |  |
| **Animal #** | **Control Vehicle** | **DNCB Solution** | | **Control**  **Vehicle** | **DNCB Solution** |  |
| 0682 | 0 | 1 | | 0 | 2 | + |
| 0683 | 0 | 2 | | 0 | 2 | + |
| 0684 | 0 | 2 | | 0 | 1 | + |
| 0685 | 0 | 2 | | 0 | 1 | + |
| 0686 | 0 | 1 | | 0 | 1 | + |
| 0687 | 0 | 1 | | 0 | 2 | + |
| 0688 | 0 | 1 | | 0 | 2 | + |
| 0689 | 0 | 2 | | 0 | 2 | + |
| 0690 | 0 | 2 | | 0 | 1 | + |
| 0691 | 0 | 1 | | 0 | 2 | + |
| 0692 | 0 | 2 | | 0 | 2 | + |
| **Control Group** | | | | | | **Results (+) or (-)** |
| **Animal #** | **Control**  **Vehicle** | **DNCB**  **Solution** | | **Control**  **Vehicle** | **DNCB**  **Solution** |  |
| 0676 | 0 | 0 | | 0 | 0 | - |
| 0677 | 0 | 0 | | 0 | 0 | - |
| 8129 | 0 | 0 | | 0 | 0 | - |
| 0679 | 0 | 0 | | 0 | 0 | - |
| 0680 | 0 | 0 | | 0 | 0 | - |
| 0681 | 0 | 0 | | 0 | 0 | - |

**Supplemental Table 3. Irritation test scores for the positive control group (0.15% sodium lauryl sulfate dissolved in 0.9% NS).** Injection sites were evaluated for gross evidence of erythema and edema at 24 ± 2, 48 ± 2 and 72 ± 2 h using the following grading system: 0 = no erythema, edema; 1 = very slight erythema, edema; 2 = well defined erythema, edema (edges of area well-defined by definite raising), 3 = moderate erythema, edema (raised ~1 mm); 4 = severe erythema (beet redness) to eschar formation preventing grading of erythema, severe edema (raised > 1 mm and extending beyond exposure area), according to ISO 10993-10 guidelines.

ER=Erythema; ED=Edema

| **Rabbit #**  **53921** | **Control Scores: 0.9% NS** | | | | | | | | | | **Test Scores: 0.15% sodium lauryl sulfate** | | | | | | | | | |
| --- | --- | --- | --- | --- | --- | --- | --- | --- | --- | --- | --- | --- | --- | --- | --- | --- | --- | --- | --- | --- |
|  | **24 Hour**  **ER ED** | | | **48 Hour**  **ERED** | | | **72 Hour**  **ER ED** | | | | | **24 Hour**  **ER ED** | | | **48 Hour**  **ER ED** | | | **72 Hour**  **ER ED** | | |
| Site 1 | 0 | | 0 | 0 | 0 | | 0 | | 0 | | | 1 | 0 | | 1 | 0 | | 1 | 0 | |
| Site 2 | 0 | | 0 | 0 | 0 | | 0 | | 0 | | | 1 | 0 | | 1 | 0 | | 1 | 0 | |
| Site 3 | 0 | | 0 | 0 | 0 | | 0 | | 0 | | | 2 | 1 | | 2 | 1 | | 2 | 1 | |
| Site 4 | 0 | | 0 | 0 | 0 | | 0 | | 0 | | | 2 | 2 | | 2 | 1 | | 2 | 1 | |
| Site 5 | 0 | | 0 | 0 | 0 | | 0 | | 0 | | | 2 | 2 | | 2 | 1 | | 1 | 1 | |
| Total | 0 | | | 0 | | | 0 | | | | | 13 | | | 11 | | | 10 | | |
| **Rabbit#** | **Control Scores** | | | | | | | | | | **Test Scores** | | | | | | | | | |
| **53922** | **24 Hour ER ED** | | | **48 Hour ER ED** | | | | **72 Hour ER ED** | | | **24 Hour ER ED** | | | | **48 Hour ER ED** | | | **72 Hour ER ED** | | |
| Site 1 | 0 | 0 | | 0 | | 0 | | 0 | | 0 | 2 | | | 1 | 1 | | 0 | 1 | | 0 |
| Site 2 | 0 | 0 | | 0 | | 0 | | 0 | | 0 | 2 | | | 1 | 1 | | 0 | 1 | | 0 |
| Site 3 | 0 | 0 | | 0 | | 0 | | 0 | | 0 | 2 | | | 1 | 1 | | 0 | 2 | | 0 |
| Site 4 | 0 | 0 | | 0 | | 0 | | 0 | | 0 | 2 | | | 1 | 1 | | 0 | 1 | | 0 |
| Site 5 | 0 | 0 | | 0 | | 0 | | 0 | | 0 | 2 | | | 2 | 1 | | 0 | 1 | | 0 |
| Total | 0 | | | 0 | | | | 0 | | | 16 | | | | 5 | | | 6 | | |
| **Rabbit #** | **Control Scores** | | | | | | | | | | **Test Scores** | | | | | | | | | |
| **53923** | **24 Hour ER ED** | | | **48 Hour ER ED** | | | | **72 Hour ER ED** | | | **24 Hour ER ED** | | | | **48 Hour ER ED** | | | **72 Hour ER ED** | | |
| Site 1 | 0 | 0 | | 0 | | 0 | | 0 | | 0 | 1 | | | 1 | 2 | | 2 | 1 | | 0 |
| Site 2 | 0 | 0 | | 0 | | 0 | | 0 | | 0 | 1 | | | 1 | 2 | | 1 | 2 | | 0 |
| Site 3 | 0 | 0 | | 0 | | 0 | | 0 | | 0 | 1 | | | 2 | 1 | | 2 | 2 | | 1 |
| Site 4 | 0 | 0 | | 0 | | 0 | | 0 | | 0 | 1 | | | 2 | 2 | | 2 | 2 | | 1 |
| Site 5 | 0 | 0 | | 0 | | 0 | | 0 | | 0 | 1 | | | 2 | 1 | | 2 | 2 | | 1 |
| Total | 0 | | | 0 | | | | 0 | | | 13 | | | | 17 | | | 12 | | |
| **Rabbit #** | **Control Scores (Total ER & ED)** | | | | | | | | | | **Test Scores (Total ER & ED)** | | | | | | | | | |
| 53921 | 0 | | | 0 | | | | 0 | | | 13 | | | | 11 | | | 10 | | |
| 53922 | 0 | | | 0 | | | | 0 | | | 16 | | | | 5 | | | 6 | | |
| 53923 | 0 | | | 0 | | | | 0 | | | 13 | | | | 17 | | | 12 | | |
| **Rabbit #** | **Total / 15** | | | | | | | | | | **Total / 15** | | | | | | | | | |
| 53921 | 0 | | | | | | | | | | 2.3 | | | | | | | | | |
| 53922 | 0 | | | | | | | | | | 1.8 | | | | | | | | | |
| 53923 | 0 | | | | | | | | | | 2.8 | | | | | | | | | |
| **Average (Total / 3)** | 0 / 3 = 0 | | | | | | | | | | 6.9 / 3 = 2.3 | | | | | | | | | |
| **Comparative Results (Average Test – Average Control)** | | | | | | 2.3 – 0 = **2.3** | | | | | | | | | | | | | | |

**Supplemental Table 4. Irritation test scores for the test group for test article extracted in 0.9 % NS as the vehicle.** Injection sites were evaluated for gross evidence of erythema and edema at 24 ± 2, 48 ± 2 and 72 ± 2 h using the following grading system: 0 = no erythema, edema; 1 = very slight erythema, edema; 2 = well defined erythema, edema (edges of area well-defined by definite raising), 3 = moderate erythema, edema (raised ~1 mm); 4 = severe erythema (beet redness) to eschar formation preventing grading of erythema, severe edema (raised > 1 mm and extending beyond exposure area), according to ISO 10993-10 guidelines.

ER=Erythema; ED=Edema

| **Rabbit #**  **51447** | **Control Scores: 0.9% NS** | | | | | | **Test Scores: test article extract in 0.9% NS** | | | | | |
| --- | --- | --- | --- | --- | --- | --- | --- | --- | --- | --- | --- | --- |
|  | **24 Hour**  **ER ED** | | **48 Hour**  **ER ED** | | **72 Hour**  **ER ED** | | **24 Hour**  **ER ED** | | **48 Hour**  **ER ED** | | **72 Hour**  **ER ED** | |
| Site 1 | 0 | 0 | 0 | 0 | 0 | 0 | 0 | 0 | 0 | 0 | 0 | 0 |
| Site 2 | 0 | 0 | 0 | 0 | 0 | 0 | 0 | 0 | 0 | 0 | 0 | 0 |
| Site 3 | 0 | 0 | 0 | 0 | 0 | 0 | 0 | 0 | 0 | 0 | 0 | 0 |
| Site 4 | 0 | 0 | 0 | 0 | 0 | 0 | 0 | 0 | 0 | 0 | 0 | 0 |
| Site 5 | 0 | 0 | 0 | 0 | 0 | 0 | 0 | 0 | 0 | 0 | 0 | 0 |
| Total | 0 | | 0 | | 0 | | 0 | | 0 | | 0 | |
| **Rabbit #** | **Control Scores** | | | | | | **Test Scores** | | | | | |
| **51448** | **24 Hour**  **ER ED** | | **48 Hour**  **ER ED** | | **72 Hour**  **ER ED** | | **24 Hour**  **ER ED** | | **48 Hour**  **ER ED** | | **72 Hour**  **ER ED** | |
| Site 1 | 0 | 0 | 0 | 0 | 0 | 0 | 0 | 0 | 0 | 0 | 0 | 0 |
| Site 2 | 0 | 0 | 0 | 0 | 0 | 0 | 0 | 0 | 0 | 0 | 0 | 0 |
| Site 3 | 0 | 0 | 0 | 0 | 0 | 0 | 0 | 0 | 0 | 0 | 0 | 0 |
| Site 4 | 0 | 0 | 0 | 0 | 0 | 0 | 0 | 0 | 0 | 0 | 0 | 0 |
| Site 5 | 0 | 0 | 0 | 0 | 0 | 0 | 0 | 0 | 0 | 0 | 0 | 0 |
| Total | 0 | | 0 | | 0 | | 0 | | 0 | | 0 | |
| **Rabbit #** | **Control Scores** | | | | | | **Test Scores** | | | | | |
| **51449** | **24 Hour**  **ER ED** | | **48 Hour**  **ER ED** | | **72 Hour**  **ER ED** | | **24 Hour**  **ER ED** | | **48 Hour**  **ER ED** | | **72 Hour**  **ER ED** | |
| Site 1 | 0 | 0 | 0 | 0 | 0 | 0 | 0 | 0 | 0 | 0 | 0 | 0 |
| Site 2 | 0 | 0 | 0 | 0 | 0 | 0 | 0 | 0 | 0 | 0 | 0 | 0 |
| Site 3 | 0 | 0 | 0 | 0 | 0 | 0 | 0 | 0 | 0 | 0 | 0 | 0 |
| Site 4 | 0 | 0 | 0 | 0 | 0 | 0 | 0 | 0 | 0 | 0 | 0 | 0 |
| Site 5 | 0 | 0 | 0 | 0 | 0 | 0 | 0 | 0 | 0 | 0 | 0 | 0 |
| Total | 0 | | 0 | | 0 | | 0 | | 0 | | 0 | |
| **Rabbit #** | **Control Scores (Total ER & ED)** | | | | | | **Test Scores (Total ER & ED)** | | | | | |
| 51447 | 0 | | 0 | | 0 | | 0 | | 0 | | 0 | |
| 51448 | 0 | | 0 | | 0 | | 0 | | 0 | | 0 | |
| 51449 | 0 | | 0 | | 0 | | 0 | | 0 | | 0 | |
| **Rabbit #** | **Total / 15** | | | | | | **Total / 15** | | | | | |
| 51447 | 0 | | | | | | 0 | | | | | |
| 51448 | 0 | | | | | | 0 | | | | | |
| 51449 | 0 | | | | | | 0 | | | | | |
| **Average (Total / 3)** | 0 / 3 = 0 | | | | | | 0 / 3 = 0 | | | | | |
| **Comparative Results (Average Test – Average Control)** | | | | 0 – 0 = **0** | | | | | | | | |

**Supplemental Table 5. Irritation test scores for the test group for test article extracted in SO as the vehicle.** Injection sites were evaluated for gross evidence of erythema and edema at 24 ± 2, 48 ± 2 and 72 ± 2 h using the following grading system: 0 = no erythema, edema; 1 = very slight erythema, edema; 2 = well defined erythema, edema (edges of area well-defined by definite raising), 3 = moderate erythema, edema (raised ~1 mm); 4 = severe erythema (beet redness) to eschar formation preventing grading of erythema, severe edema (raised > 1 mm and extending beyond exposure area), according to ISO 10993-10 guidelines.

ER=Erythema; ED=Edema

| **Rabbit #**  **51447** | **Control Scores: SO** | | | | | | **Test Scores: Test article extract in SO** | | | | | |
| --- | --- | --- | --- | --- | --- | --- | --- | --- | --- | --- | --- | --- |
|  | **24 Hour**  **ER ED** | | **48 Hour**  **ER ED** | | **72 Hour**  **ER ED** | | **24 Hour**  **ER ED** | | **48 Hour**  **ER ED** | | **72 Hour**  **ER ED** | |
| Site 1 | 2 | 0 | 1 | 0 | 1 | 0 | 2 | 0 | 1 | 0 | 1 | 0 |
| Site 2 | 2 | 0 | 1 | 0 | 1 | 0 | 2 | 0 | 2 | 0 | 2 | 0 |
| Site 3 | 2 | 0 | 1 | 0 | 1 | 0 | 2 | 0 | 1 | 0 | 1 | 0 |
| Site 4 | 2 | 0 | 1 | 0 | 0 | 0 | 2 | 0 | 1 | 0 | 2 | 0 |
| Site 5 | 2 | 0 | 1 | 0 | 1 | 0 | 2 | 0 | 1 | 0 | 1 | 0 |
| Total | 10 | | 5 | | 4 | | 10 | | 6 | | 7 | |
| **Rabbit #** | **Control Scores** | | | | | | **Test Scores** | | | | | |
| **51448** | **24 Hour**  **ER ED** | | **48 Hour**  **ER ED** | | **72 Hour**  **ER ED** | | **24 Hour**  **ER ED** | | **48 Hour**  **ER ED** | | **72 Hour**  **ER ED** | |
| Site 1 | 2 | 0 | 1 | 0 | 1 | 0 | 2 | 0 | 1 | 0 | 1 | 0 |
| Site 2 | 2 | 0 | 1 | 0 | 1 | 0 | 2 | 0 | 1 | 0 | 1 | 0 |
| Site 3 | 2 | 0 | 1 | 0 | 1 | 0 | 2 | 0 | 1 | 0 | 0 | 0 |
| Site 4 | 2 | 0 | 1 | 0 | 1 | 0 | 2 | 0 | 1 | 0 | 1 | 0 |
| Site 5 | 2 | 0 | 1 | 0 | 1 | 0 | 2 | 0 | 2 | 0 | 2 | 0 |
| Total | 10 | | 5 | | 5 | | 10 | | 6 | | 5 | |
| **Rabbit #** | **Control Scores** | | | | | | **Test Scores** | | | | | |
| **51449** | **24 Hour**  **ER ED** | | **48 Hour**  **ER ED** | | **72 Hour**  **ER ED** | | **24 Hour**  **ER ED** | | **48 Hour**  **ER ED** | | **72 Hour**  **ER ED** | |
| Site 1 | 2 | 0 | 1 | 0 | 1 | 0 | 1 | 0 | 1 | 0 | 1 | 0 |
| Site 2 | 2 | 0 | 1 | 0 | 1 | 0 | 1 | 0 | 1 | 0 | 1 | 0 |
| Site 3 | 2 | 0 | 1 | 0 | 1 | 0 | 2 | 0 | 1 | 0 | 1 | 0 |
| Site 4 | 2 | 0 | 1 | 0 | 1 | 0 | 2 | 0 | 1 | 0 | 1 | 0 |
| Site 5 | 2 | 0 | 1 | 0 | 1 | 0 | 2 | 0 | 1 | 0 | 1 | 0 |
| Total | 10 | | 5 | | 5 | | 8 | | 5 | | 5 | |
| **Rabbit #** | **Control Scores (Total ER & ED)** | | | | | | **Test Scores (Total ER & ED)** | | | | | |
| 51447 | 10 | | 5 | | 4 | | 10 | | 6 | | 7 | |
| 51448 | 10 | | 5 | | 5 | | 10 | | 6 | | 5 | |
| 51449 | 10 | | 5 | | 5 | | 8 | | 5 | | 5 | |
| **Rabbit #** | **Total / 15** | | | | | | **Total / 15** | | | | | |
| 51447 | 1.3 | | | | | | 1.5 | | | | | |
| 51448 | 1.3 | | | | | | 1.4 | | | | | |
| 51449 | 1.3 | | | | | | 1.2 | | | | | |
| **Average (Total / 3)** | 3.9 / 3 = 1.3 | | | | | | 4.1 / 3 = 1.4 | | | | | |
| **Comparative Results (Average Test – Average Control)** | | | | 1.4 – 1.3 = **0.1** | | | | | | | | |

**Supplemental Table 6. Pyrogenicity test data.** Animal weight, dose (test article extract in 0.9% NS) and temperature measurements for all three animals used in this test.

| **Rabbit #** | **52889** | **52891** | **51215** |
| --- | --- | --- | --- |
| Weight (kg) | 3.2 | 3.7 | 3.2 |
| Dose (mL) | 32 | 37 | 32 |
| **Baseline Temp (**°C**)** | **39.1** | **39.6** | **39.7** |
| 1.0 h | 39.0 | 39.4 | 39.5 |
| 1.5 h | 39.1 | 39.5 | 39.5 |
| 2.0 h | 39.2 | 39.4 | 39.4 |
| 2.5 h | 39.2 | 39.4 | 39.4 |
| 3.0 h | 39.3 | 39.6 | 39.4 |
| **Max Temp Rise (**°C**)** | **0.2** | **0 ^a^** | **0^a^** |

**^a^** A negative value is reported as zero (0) temperature rise.

**Supplemental Table 7. Implantation test data for animals implanted with the test article.** Semiquantitative scoring of tissue from animals implanted with the test article electrodes (n=5 sheep). The tissue was assessed for accumulation of immune system cells (polymorphonuclear cell, lymphocytes, plasma cells, neutrophils, lymphocytes, macrophages/gitter cells, multinucleated giant cells), neo-vascularization, fibrosis, astrocytosis/fatty infiltration, according to the criterial listed in ISO 10993-6:2016.

| **Scored attributes** | **Test Article Site Scores** | | | | | | | | | |
| --- | --- | --- | --- | --- | --- | --- | --- | --- | --- | --- |
| **Animal** | **18S0136** | | **18S0141** | | **18S0151** | | **18S0153** | | **18S0155** | |
| **Site #** | 1 | 2 | 1 | 2 | 1 | 2 | 1 | 2 | 1 | 2 |
| **Cell type/response** |  |  |  |  |  |  |  |  |  |  |
| Polymorphonuclear cells | 0 | 3 | 0 | 0 | 0 | 0 | 0 | 0 | 0 | 0 |
| Lymphocytes | 1 | 2 | 0 | 0 | 0 | 0 | 0 | 0 | 1 | 1 |
| Plasma cells | 0 | 1 | 0 | 0 | 0 | 0 | 0 | 0 | 0 | 0 |
| Macrophages/gitter cells | 1 | 2 | 1 | 1 | 0 | 0 | 0 | 0 | 1 | 0 |
| Multinucleated giant cells | 0 | 1 | 0 | 0 | 0 | 0 | 0 | 0 | 0 | 0 |
| Necrosis | 0 | 0 | 0 | 0 | 0 | 0 | 0 | 0 | 0 | 0 |
| **Cell type/response total** | 2 | 9 | 1 | 1 | 0 | 0 | 0 | 0 | 2 | 1 |
| **A=[Cell type/response subtotal] x 2** | 4 | 18 | 2 | 2 | 0 | 0 | 0 | 0 | 4 | 2 |
| **Tissue response** |  |  |  |  |  |  |  |  |  |  |
| Neovascularization | 0 | 1 | 0 | 0 | 0 | 0 | 0 | 0 | 0 | 0 |
| Fibrosis | 0 | 1 | 0 | 0 | 0 | 0 | 0 | 0 | 0 | 0 |
| Astrocytosis/Fatty infiltration | 0 | 0 | 0 | 0 | 0 | 0 | 0 | 0 | 0 | 0 |
| **B= Tissue response subtotal** | 0 | 2 | 0 | 0 | 0 | 0 | 0 | 0 | 0 | 0 |
| **A + B = Test Site Total Score** | 4 | 20 | 2 | 2 | 0 | 0 | 0 | 0 | 4 | 2 |
| **Test Group Total Score** | 34 | | | | | | | | | |
| **Number of Sites Scored** | 10 | | | | | | | | | |
| **Test Group Average Score** | **[34/10] = 3.4** | | | | | | | | | |

**Supplemental Table 8. Implantation test data for animals implanted with the control article (USP HDPE).** Semiquantitative scoring of tissue from animals implanted with the control article (n=4 sheep). The tissue was assessed for accumulation of immune system cells (polymorphonuclear cell, lymphocytes, plasma cells, neutrophils, lymphocytes, macrophages/gitter cells, multinucleated giant cells), neo-vascularization, fibrosis, astrocytosis/fatty infiltration, according to the criterial listed in ISO 10993-6:2016.

| **Scored attributes** | **Control Article Site Scores** | | | | | | | | | | | | | |  |
| --- | --- | --- | --- | --- | --- | --- | --- | --- | --- | --- | --- | --- | --- | --- | --- |
| **Animal** | **18S0133** | | | **18S0148** | | | **18S0152** | | | | **18S0154** | | | |  |
| **Site #** | 1 | 2 | 3 | 1 | 2 | 3 | | 1 | 2 | 3 | | 1 | 2 | 3 | |
| **Cell type/response** |  |  |  |  |  |  | |  |  |  | |  |  |  | |
| Polymorphonuclear cells | 0 | 0 | 0 | 0 | 0 | 0 | | 1 | 0 | 0 | | 0 | 0 | 0 | |
| Lymphocytes | 0 | 0 | 0 | 0 | 0 | 0 | | 0 | 1 | 0 | | 0 | 0 | 1 | |
| Plasma cells | 0 | 0 | 0 | 0 | 0 | 0 | | 0 | 0 | 0 | | 0 | 0 | 0 | |
| Macrophages/gitter cells | 1 | 1 | 0 | 0 | 0 | 0 | | 0 | 1 | 0 | | 0 | 1 | 1 | |
| Multinucleated giant cells | 0 | 0 | 0 | 0 | 0 | 0 | | 1 | 0 | 0 | | 1 | 0 | 0 | |
| Necrosis | 0 | 0 | 0 | 0 | 0 | 0 | | 0 | 0 | 0 | | 0 | 0 |  | |
| **Cell type/response total** | 1 | 1 | 0 | 0 | 0 | 0 | | 2 | 2 | 0 | | 1 | 1 | 2 | |
| **A = [Cell type/response subtotal] x 2** | 2 | 2 | 0 | 0 | 0 | 0 | | 4 | 4 | 0 | | 2 | 2 | 4 | |
| **Tissue response** |  |  |  |  |  |  | |  |  |  | |  |  |  | |
| Neovascularization | 0 | 0 | 0 | 0 | 0 | 0 | | 0 | 0 | 0 | | 0 | 0 | 0 | |
| Fibrosis | 0 | 0 | 0 | 0 | 0 | 0 | | 0 | 0 | 0 | | 0 | 0 | 0 | |
| Astrocytosis/Fatty infiltration | 0 | 0 | 0 | 0 | 0 | 0 | | 0 | 0 | 0 | | 0 | 0 | 0 | |
| **B = Tissue response subtotal** | 0 | 0 | 0 | 0 | 0 | 0 | | 0 | 0 | 0 | | 0 | 0 | 0 | |
| **A + B = Test Site Total Score** | 0 | 0 | 0 | 0 | 0 | 0 | | 0 | 0 | 0 | | 0 | 0 | 0 | |
| **Test Group Total Score** | 20 | | | | | | | | | | | | | |  |
| **Number of Sites Scored** | 12 | | | | | | | | | | | | | |  |
| **Test Group Average Score** | **[20/12] = 1.7** | | | | | | | | | | | | | |  |

**References:**

Magnusson, B., and Kligman, A.M. (1969). The identification of contact allergens by animal assay. The guinea pig maximization test. *J Invest Dermatol* 52(3)**,** 268-276. doi: 10.1038/jid.1969.42.

Schlede, E., and Eppler, R. (1995). Testing for skin sensitization according to the notification procedure for new chemicals: the Magnusson and Kligman test. *Contact Dermatitis* 32(1)**,** 1-4. doi: 10.1111/j.1600-0536.1995.tb00830.x.
